# Histone Lactylation Is Involved in Mouse Oocyte Maturation and Embryo Development

**DOI:** 10.3390/ijms25094821

**Published:** 2024-04-28

**Authors:** Diqi Yang, Haoyi Zheng, Wenjie Lu, Xueqi Tian, Yanyu Sun, Hui Peng

**Affiliations:** 1 School of Tropical Agriculture and Forestry, Hainan University, Haikou 570228, China; yangdiqi@hainanu.edu.cn (D.Y.);; 2 College of Animal Science, Fujian Agriculture and Forestry University, Fuzhou 350002, China

**Keywords:** post-translational modifications, histone lactylation, oocyte, embryo, lactate

## Abstract

Numerous post-translational modifications are involved in oocyte maturation and embryo development. Recently, lactylation has emerged as a novel epigenetic modification implicated in the regulation of diverse cellular processes. However, it remains unclear whether lactylation occurs during oocyte maturation and embryo development processes. Herein, the lysine lactylation (Kla) modifications were determined during mouse oocyte maturation and early embryo development by immunofluorescence staining. Exogenous lactate was supplemented to explore the consequences of modulating histone lactylation levels on oocyte maturation and embryo development processes by transcriptomics. Results demonstrated that lactylated proteins are widely present in mice with tissue- and cell-specific distribution. During mouse oocyte maturation, immunofluorescence for H3K9la, H3K14la, H4K8la, and H4K12la was most intense at the germinal vesicle (GV) stage and subsequently weakened or disappeared. Further, supplementing the culture medium with 10 mM sodium lactate elevated both the oocyte maturation rate and the histone Kla levels in GV oocytes, and there were substantial increases in Kla levels in metaphase II (MII) oocytes. It altered the transcription of molecules involved in oxidative phosphorylation. Moreover, histone lactylation levels changed dynamically during mouse early embryogenesis. Sodium lactate at 10 mM enhanced early embryo development and significantly increased lactylation, while impacting glycolytic gene transcription. This study reveals the roles of lactylation during oocyte maturation and embryo development, providing new insights to improving oocyte maturation and embryo quality.

## 1. Introduction

The process of oocyte maturation is a unique form of cell division that sets it apart from mitosis. It represents the reactivation and culmination of the initial meiotic division [1]. Oocytes remain in a state of arrest at the germinal vesicle (GV) stage for many years. Upon stimulation by a surge of luteinizing hormone (LH), there is an occurrence of germinal vesicle breakdown (GVBD) with a gradual compaction of chromosomes [2]. Once chromosomal condensation is complete, oocytes progress into metaphase I (MI), where chromosomes align along the metaphase plate, and spindle microtubules attach to kinetochores, laying the foundation for the subsequent segregation of chromosomes during anaphase I [3]. Following the expulsion of the first polar body, oocytes reach a state of arrest at metaphase II (MII) until fertilization occurs. Any aberrations in this intricate process may result in meiotic arrest and, consequently, fertilization failure. Considering the absence of transcriptional activity during oocyte maturation, the significance of post-translational modifications (PTMs) becomes evident in facilitating the successful completion of oocyte maturation [4]. Thus, PTMs play critical roles during oocyte maturation.

Following fertilization, the zygote initiates the expression of genes specific to embryonic development, and this expression pattern undergoes dynamic changes throughout the preimplantation development phase. Maternal mRNAs and proteins that originate during oogenesis exert control over virtually all aspects of the initial embryonic development, even though the transcription of the zygotic genome remains quiescent [5]. These proteins are primarily activated at the moment of fertilization and during the maternal-to-zygotic transition. Zygotic genome activation is predominantly observed at the 2-cell stage in mice, while in other species, it occurs at the 4-cell and 8-cell stages [6]. Because the zygotic genome remains dormant during the early stages of embryogenesis, the significance of post-translational modifications (PTMs) becomes particularly evident. Recent research has demonstrated that PTMs play a crucial role in eliminating maternal mRNAs and degrading proteins [7]. Deviations in PTMs during both oocyte maturation and the early stages of embryo development may lead to complications such as implantation failure and fetal abnormalities [8,9].

Numerous post-translational modifications are involved in the processes of oocyte growth and maturation, encompassing acetylation, methylation, phosphorylation, ubiquitination, and SUMOylation of a variety of proteins. These modifications play roles of varying significance in the regulation of chromatin structure and gene expression [10,11,12]. Recent investigations have unveiled a novel function of lactate in orchestrating the shift from inflammatory to reparative macrophages and instigating the expression of homeostatic genes through histone lysine lactylation, thereby upholding immune equilibrium [13,14]. While previous studies have revealed the role of Tfap2a in regulating mouse oocyte maturation and its effects on histone acetylation and lactylation levels [15], the specific functions and dynamic changes of lactylation during oocyte maturation and early embryo development processes remain to be explored in depth. Currently, there is a lack of systematic studies investigating the roles of histone lactylation in regulating these critical biological processes. Moreover, Yang et al. demonstrated the presence of histone lactylation marks in mouse oocytes and preimplantation embryos, as well as the potential impact of hypoxic conditions on lactylation levels and embryo development [16]; a comprehensive understanding of the regulatory roles of lactylation during these critical developmental stages is still lacking.

Therefore, in this study, we first examined the expression and distribution of lactylated proteins in various mouse tissues to confirm that lactylation is a prevalent post-translational modification. We then focused on investigating the expression patterns and dynamic changes in histone lactylation during the maturation of mouse oocytes and the early embryo development of using immunofluorescence staining with pan-lactylation and site-specific antibodies. Additionally, we supplemented exogenous lactate to oocytes and embryos to explore the consequences of modulating histone lactylation levels on oocyte maturation and embryo development processes. Furthermore, we analyzed the impact of enhanced lactylation on the transcriptome of MII oocytes and 2-cell embryos.

## 2. Results

### 2.1. Localization of Kla-Modified Proteins in Various Tissues of Mice

**Figure 1:**
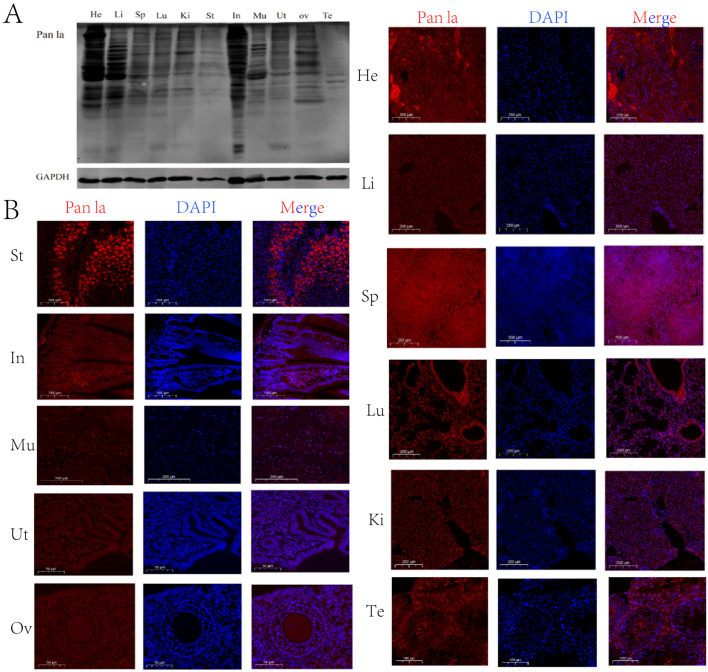
Localization of lysine-lactylated (Kla) proteins in various tissues of mice. (**A**) Immunoblotting analysis of pan-lactylation levels in different mouse tissues. (**B**) Immunofluorescence staining showing localization of lactylated proteins in multiple mouse tissues including heart (He), liver (Li), spleen (Sp), lung (Lu), kidney (Ki), stomach (St), intestine (In), muscle (Mu), uterus (Ut), ovary (Ov), and testis (Te).

To investigate the expression of lactylated proteins in mouse tissues, we performed immunoblotting to examine lactylation levels in various mouse tissues. As shown in Figure 1A, multiple bands were detected in the heart, liver, spleen, lung, kidney, stomach, intestine, muscle, uterus, ovary, and testis, indicating lactylation of diverse proteins in different mouse tissues. To further validate these results, we used immunofluorescence to characterize the localization of lactylated proteins in the heart, liver, spleen, lung, kidney, stomach, small intestine, muscle, uterus, ovary, and testis tissues. In cardiac tissues, lactylated proteins were detected primarily in pacemaker cells, connective tissues, and ganglia (Figure 1B). The hepatocellular nucleus and cytoplasm showed strong lactylated protein signals in the liver (Figure 1B). The spleen displayed marked lactylation in trabeculae, white pulp and red pulp regions (Figure 1B). In the lung, alveolar epithelial cells and basement membranes were the major sites of lactylated protein localization (Figure 1B). Glomeruli, proximal convoluted tubules, and distal convoluted tubules exhibited substantial lactylation in the kidney (Figure 1B). In the stomach, lactylated proteins were abundant in the serosa, muscularis, submucosa, and mucosa layers (Figure 1B). The small intestine featured lactylated proteins mainly in its mucosa, submucosa, muscularis, and serosa layers (Figure 1B). Lactylated proteins were found concentrated in the nucleus and cytoplasm of myocytes in muscle tissues (Figure 1B). Oocytes, stromal cells, and granulosa cells displayed lactylation in the ovary, with the most pronounced signals in the oocyte cytoplasm (Figure 1B). The mouse uterus showed lactylated protein localization primarily in the serosa, myometrium, and endometrium, with the highest levels in the endometrium (Figure 1B). In testicular tissues, lactylated proteins were present largely in spermatogenic cells and interstitial cells, with enriched localization in the interstitial cells (Figure 1B).

### 2.2. Lactylation of Histones Occurred during Mouse Oocyte Maturation

**Figure 2:**
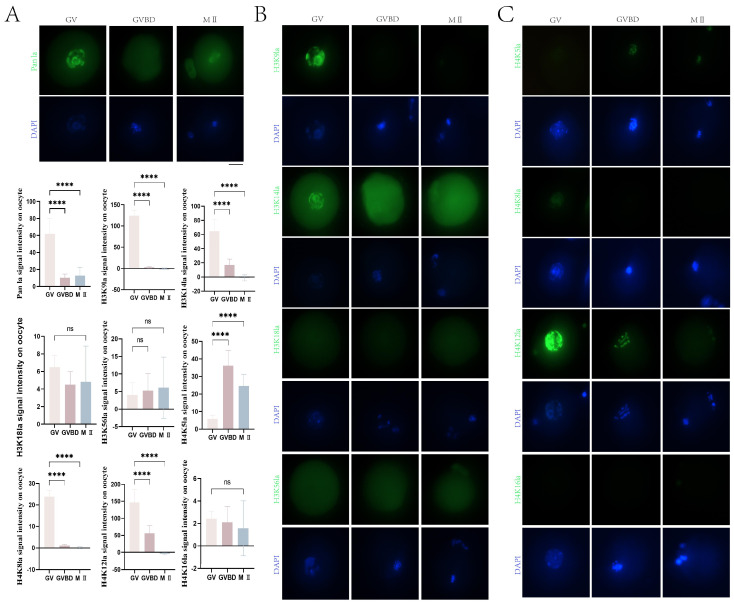
Histone lactylation during mouse oocyte maturation. (**A**) Immunofluorescence analysis of pan-lactylation levels in germinal vesicle (GV), germinal vesicle breakdown (GVBD), and metaphase II (MII) stage mouse oocytes. DNA counterstained with DAPI (blue). Scale bar = 10 μm. (**B**) Immunofluorescence analysis of histone H3 lysine lactylation (Kla) during mouse oocyte maturation using antibodies against H3K9la, H3K14la, H3K18la, and H3K56la. (**C**) Immunofluorescence analysis of histone H4 Kla during mouse oocyte maturation using antibodies against H4K5la, H4K8la, H4K12la, and H4K16la. DNA counterstained with DAPI (blue). Scale bar = 10 μm. Fluorescence intensities were quantified and are presented as mean ± SD (**** *p* < 0.0001; ns *p* > 0.05).

To examine whether lactylation takes place and its dynamic changes during mouse oocyte maturation, we performed immunofluorescence staining using a pan-lactylation antibody in GV, GVBD, and MII stage oocytes. As shown in Figure 2A, lactylation was detected in mouse oocytes at the GV, GVBD, and MII stages. GV oocytes displayed the highest level of lactylation, which was extremely significantly different from that in GVBD (6.0-fold higher, *p* < 0.01) and MII oocytes (4.6-fold higher, *p* < 0.01). Lactylation rapidly declined as mouse oocytes resumed meiosis. A slight increase in lactylation was observed as oocytes matured, but the overall level remained low.

To further examine lactylation levels at different sites of H3 during mouse oocyte maturation, we performed immunofluorescence staining using antibodies against H3K9la, H3K14la, H3K18la, and H3K56la in collected GV, GVBD, and MII oocytes. As illustrated in Figure 2B, H3K9la fluorescence signals were detected in GV oocytes, with significantly decreased fluorescence intensity in GVBD (*p* < 0.01) and MII oocytes (*p* < 0.01) compared to GV oocytes. Intense H3K14la fluorescence was present in GV oocytes and then gradually declined, with extremely significant differences between GV and GVBD/MII (*p* < 0.01) oocytes (Figure 2B). However, scarce H3K18la and H3K56la fluorescence was observed at the GV, GVBD, or MII stages (Figure 2B). We further inspected the lactylation levels at specific sites of H4 during mouse oocyte maturation. H4K5la fluorescence changed from low to high and then decreased again from the GV to GVBD (6.1-fold higher than GV, *p* < 0.01) to MII stages (4.1-fold higher than GV, *p* < 0.01), with the highest signals in GVBD oocytes that were extremely significantly different from those in GV oocytes (Figure 2C). Fluorescence in MII oocytes was also extremely significantly different from that in GV oocytes. As shown in Figure 2C, H4K8la fluorescence was detectable in GV but not GVBD or MII oocytes, with extreme significance between GV and GVBD/MII oocytes (*p* < 0.01). H4K12la fluorescence was present in GV and GVBD oocytes and gradually diminished to undetectable levels with oocyte maturation (Figure 2C). No H4K16la fluorescence was observed at the GV, GVBD, or MII stages (Figure 2C).

### 2.3. Effects of Increasing Histone Kla Levels on Mouse Oocyte Maturation

**Figure 3:**
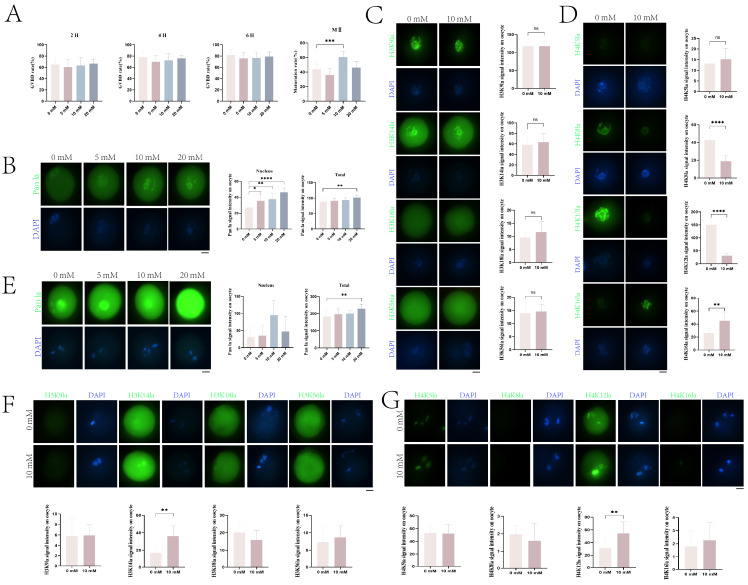
Effects of sodium lactate on histone lysine lactylation and maturation in mouse oocytes. (**A**) Effects of different sodium lactate concentrations on germinal vesicle breakdown (GVBD) at 2 h, 4 h, 6 h, and maturation rates in mice oocytes. (**B**) Immunofluorescence analysis of pan-lactylation levels in germinal vesicle (GV) phase mouse oocytes with 0 mM–20 mM sodium lactate addition. DNA counterstained with DAPI (blue). Scale bar = 10 μm. (**C**,**D**) Immunofluorescence analysis of histone H3 lysine lactylation (Kla) (**C**) and H4 Kla (**D**) in GV stage mouse oocytes treated with 0 or 10 mM sodium lactate. DNA counterstained with DAPI (blue). Scale bar = 10 μm. (**E**) Immunofluorescence analysis of histone pan-lactylation in metaphase II (MII) stage mouse oocytes treated with 0–20 mM sodium lactate. DNA counterstained with DAPI (blue). Scale bar = 10 μm. (**F**,**G**) Immunofluorescence analysis of histone H3 Kla (**F**) and H4 Kla (**G**) in MII stage mouse oocytes treated with 0 or 10 mM sodium lactate. DNA counterstained with DAPI (blue). Scale bar = 10 μm. Fluorescence intensities were quantified and presented as mean ± SD (* *p* < 0.05; ** *p* < 0.01; *** *p*  <  0.001; **** *p*  <  0.0001).

Previous studies showed that adding sodium lactate to the culture medium can enhance histone Kla modification [17]. To investigate the effects of sodium lactate on the resumption of meiosis and maturation in mouse oocytes, we supplemented the medium with 0, 5, 10, and 20 mM sodium lactate and assessed GVBD and maturation rates at different time points. As shown in Figure 3A, the GVBD rates showed no significant difference among oocyte groups treated with different concentrations of sodium lactate. For maturation rates assessed at 14 h after releasing inhibition of GV oocytes, supplementation with 10 mM sodium lactate significantly increased oocyte maturation (mean = 60.7%) compared to the control group (mean = 44.0%) (*p* < 0.01).

To examine the effects of sodium lactate on histone lactylation levels in GV oocytes, oocytes were cultured with the four concentrations of sodium lactate (0, 5, 10, 20 mM) in medium containing IBMX for 24 h, then directly fixed for immunofluorescence imaging and analysis. We observed clear pan-lactylation fluorescence signals in the nucleus of oocytes under 0, 5, 10, and 20 mM sodium lactate, with fluorescence intensity increasing significantly with sodium lactate concentration (Figure 3B). To further investigate the effects of sodium lactate on the lactylation of H3 and H4 during mouse oocyte maturation, we compared the 10 mM sodium lactate group with the 0 mM control group and detected changes in lactylation at specific sites of H3 and H4. As shown in Figure 3C, H3K9la and H3K14la fluorescence signals were detectable in the nucleus of GV oocytes in the 0 and 10 mM groups, but with no significant difference in intensity. Faint H3K18la fluorescence was observed in the nucleus of GV oocytes in the 0 and 10 mM groups, again with no significant difference (Figure 3C). No H3K56la fluorescence was detected in the GV oocyte nucleus under 0 or 10 mM sodium lactate (Figure 3C). Moreover, no H4K5la fluorescence was observed in the GV oocyte nucleus of either group (Figure 3D). H4K8la (2.2-fold higher than 10 mM, *p* < 0.01) and H4K12la (4.7-fold higher than 10 mM, *p* < 0.01) showed bright fluorescence in the GV oocyte nucleus of both groups that diminished significantly from 0 to 10 mM (Figure 3D). H4K16la fluorescence was detectable in the GV oocyte nucleus of both groups, but in contrast to other sites, the signal was extremely significantly enhanced (1.7-fold higher than 0 mM, *p* < 0.01) with increased sodium lactate concentration (Figure 3D).

We further examined the effects of sodium lactate on lactylation levels in MII oocytes using the pan-lactylation antibody. As shown in Figure 3E, clear nuclear fluorescence was observed in MII oocytes under 0, 5, 10, and 20 mM sodium lactate, with the overall signal intensity increasing gradually. Staining with the eight site-specific lactylation antibodies showed that H3K9la, H3K18la, H3K56la, H4K8la, and H4K16la fluorescence was undetectable in the MII oocyte nucleus of the 0 and 10 mM groups. H3K14la, H4K5la, and H4K12la signals were detectable in the MII oocyte nucleus of both groups. H3K14la (2.1-fold higher than 0 mM, *p* < 0.01) and H4K12la (1.7-fold higher than 0 mM, *p* < 0.01) fluorescence was extremely significantly enhanced in MII oocytes with 10 mM sodium lactate compared to 0 mM (*p* < 0.01), while the intensity of H4K5la showed no significant change.

### 2.4. Increasing Histone Kla Levels Modify Gene Expression in the MII Oocytes

**Figure 4:**
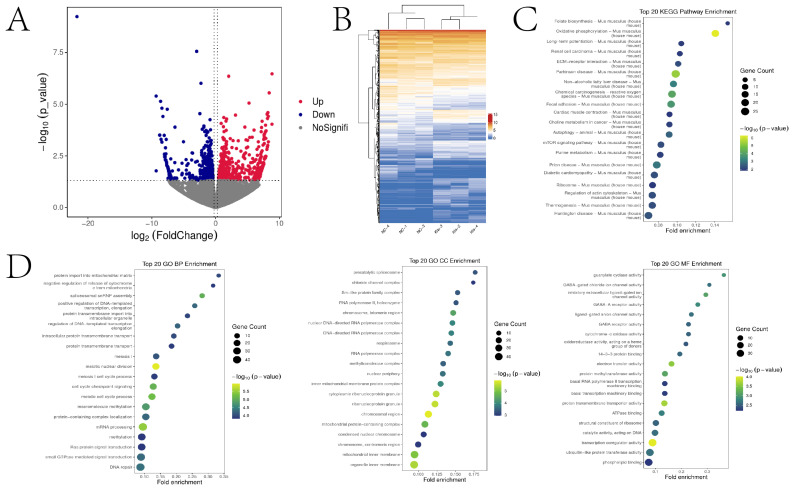
Transcriptomic changes induced by sodium lactate in mouse MII oocytes. (**A**) Volcano plot showing differentially expressed genes (DEGs). (**B**) Heatmap of DEG expression. (**C**,**D**) KEGG pathway and GO enrichment analyses of DEGs after 10 mM versus 0 mM sodium lactate treatment in mouse MII oocytes.

To elucidate the mechanisms underlying the effects of lactylation on MII oocytes, we performed transcriptomic analysis of MII oocytes treated with 10 mM sodium lactate compared to controls (CON). The results demonstrated 496 upregulated and 487 downregulated mRNAs in the sodium lactate group versus CON (Figure 4A,B). Kyoto Encyclopedia of Genes and Genomes (KEGG) pathway analysis revealed that the differentially expressed genes (DEGs) were mainly enriched in pathways related to oxidative phosphorylation, focal adhesion, and folate biosynthesis (Figure 4C). For biological process classification, meiotic nuclear division, cell cycle checkpoint signaling, and the meiotic cell cycle process were significantly enriched (Figure 4D). At the cellular component level, these proteins were highly enriched in the chromosomal region, ribonucleoprotein granule and chromosome telomeric region (Figure 4D). The DEGs in the molecular function category were highly enriched in transcription coregulator activity, proton transmembrane transporter activity, and electron transfer activity (Figure 4D).

### 2.5. Nuclear Accumulation of Histone Lactylation in Embryos

**Figure 5:**
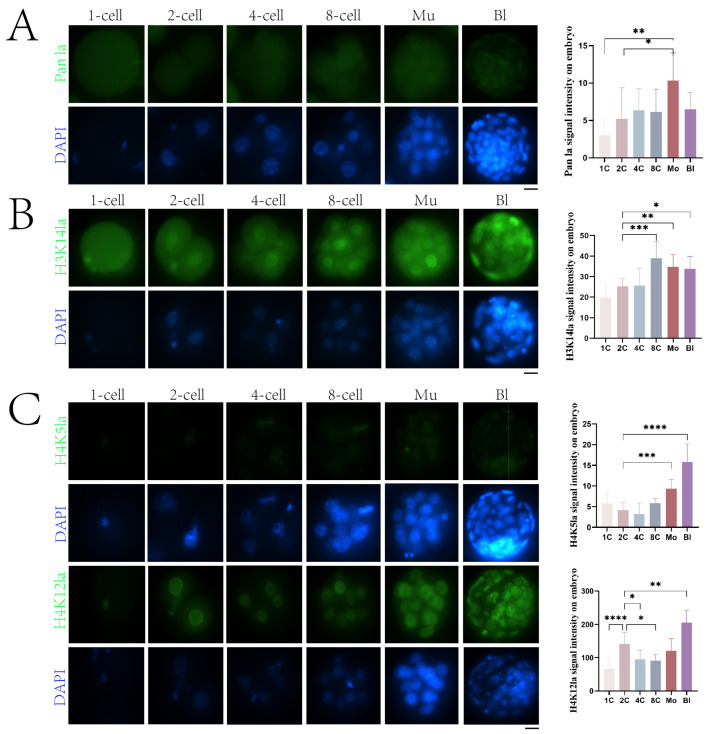
Histone lactylation during mouse early embryogenesis. (**A**) Immunofluorescence analysis of histone pan-lactylation levels at various embryonic stages. DNA counterstained with DAPI (blue). Scale bar = 10 μm. (**B**,**C**) Immunofluorescence analysis of H3 Kla (**B**) and H4 Kla (**C**) at various embryonic stages. DNA counterstained with DAPI (blue). Scale bar = 10 μm. Fluorescence intensities were quantified and presented as mean ± SD (* *p* < 0.05; ** *p* < 0.01; *** *p*  <  0.001; **** *p * <  0.0001).

To investigate lactylation levels and dynamic changes during mouse early embryo development, 1-cell, 2-cell, 4-cell, 8-cell, morula, and blastocyst stage embryos were examined by immunofluorescence intensity using a pan-lactylation antibody. As shown in Figure 5A, except for weaker fluorescence in 1-cell embryos, lactylated histones were detectable at the 2-cell, 4-cell, 8-cell, morula, and blastocyst stages. We further examined lactylation levels at different sites of H3 and H4 during mouse early development. Negligible H3K9la, H3K18la, and H3K56la fluorescence was observed at the 1-cell to blastocyst stages. In contrast, H3K14la signals were detectable at all stages examined, with lactylation at this site increasing progressively as embryos developed, peaking at the 8-cell stage and then slowly declining (Figure 5B). Additionally, H4K5la fluorescence was relatively weak at the 1-cell to 4-cell stages but gradually increased from the 8-cell to morula and blastocyst stages, with extremely significant differences between 2-cell and morula (2.2-fold higher than 2-cell, *p* < 0.01)/blastocyst embryos (3.8-fold higher than 2-cell, *p* < 0.01) (Figure 5C). H4K12la fluorescence was observable at all stages. It showed an extreme significant increase from 1-cell to 2-cell embryos (2.1-fold higher than 1-cell, *p* < 0.01), followed by a significant reduction at the 4-cell and 8-cell stages compared to 2-cell embryos (*p* < 0.05), and then a gradual re-elevation with extremely higher signals in blastocysts versus 2-cell embryos (*p* < 0.01) (Figure 5C). However, scarce H4K8la and H4K16la fluorescence was detected at any of the embryonic stages examined.

### 2.6. Increased Histone Lactylation Promotes Mouse Early Embryo Development

**Figure 6:**
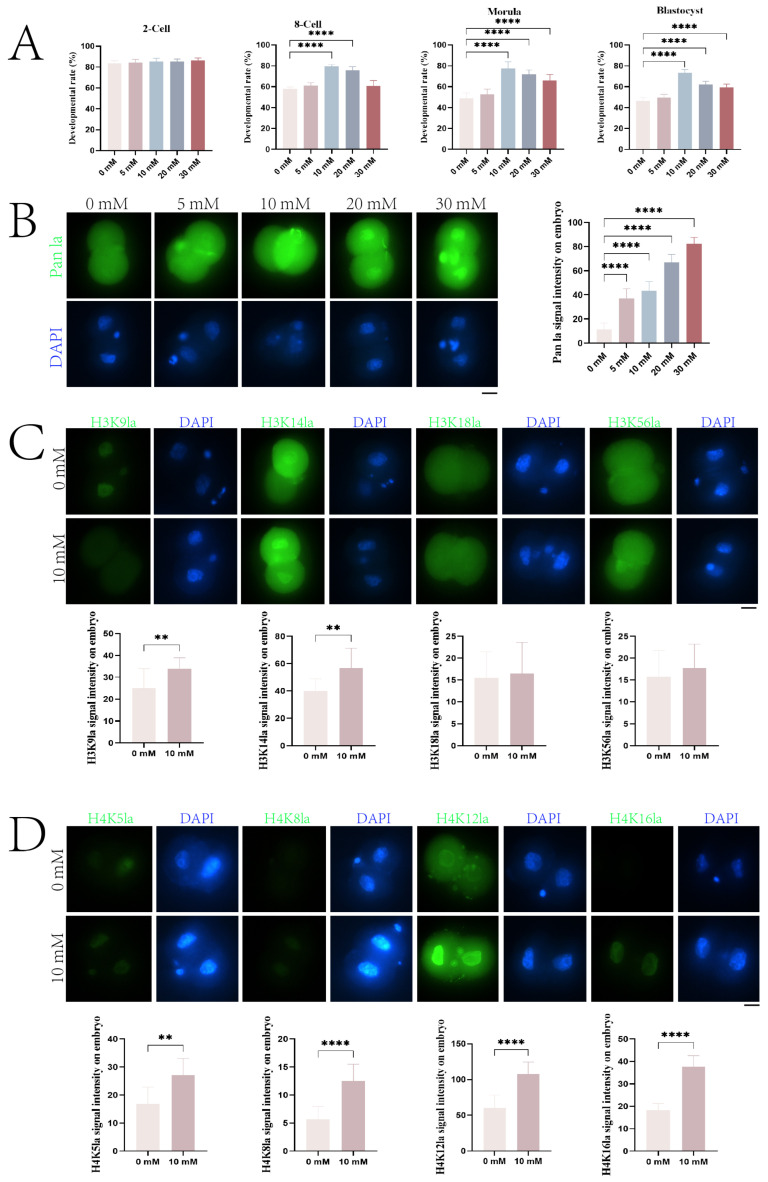
Sodium lactate enhances histone lactylation and development of mouse embryos. (**A**) Effects of sodium lactate on development rates of 2-cell, 8-cell, morula, and blastocyst stage embryos. (**B**) Immunofluorescence analysis of histone pan-lactylation in 2-cell embryos treated with 0–30 mM sodium lactate. DNA counterstained with DAPI (blue). Scale bar = 10 μm. (**C**,**D**) Immunofluorescence analysis of histone H3 Kla (**C**) and H4 Kla (**D**) in 2-cell embryos treated with 0 or 10 mM sodium lactate. DNA counterstained with DAPI (blue). Scale bar = 10 μm. Fluorescence intensities were quantified and presented as mean ± SD (** *p* < 0.01; **** *p * <  0.0001).

To investigate the effects of lactylation on mouse early embryo development, we supplemented embryo culture medium with different concentrations of sodium lactate (0, 5, 10, 20, and 30 mM). Our results showed no significant difference in 2-cell embryo development rates across the five sodium lactate concentrations (Figure 6A). For 8-cell embryos, development rates gradually increased from 0 to 10 mM sodium lactate, and then decreased from 10 to 30 mM, with the highest rate observed at 10 mM (Figure 6A). The 8-cell development rates under 10 mM (mean = 79.6%, *p* < 0.01) and 20 mM (mean = 75.7% *p* < 0.01) sodium lactate were extremely significantly higher than under 0 mM (mean = 57.7%). For morulae, development rates first increased and then decreased with escalating sodium lactate levels, peaking at 10 mM (mean = 77.6%, *p* < 0.01) (Figure 6A). Under 10, 20 (mean = 72.1%, *p* <0.01), and 30 (mean = 65.8%, *p* < 0.01) mM sodium lactate, morula development rates were extremely significantly higher than under 0 mM. Blastocyst development rates showed similar changes to the 8-cell and morula stages, rising initially and then declining, with the highest rate observed at 10 mM sodium lactate. Relative to 0 mM, blastocyst development rates were extremely significantly increased under 10 (mean = 73.1%, *p* < 0.01), 20 (mean = 62.2%, *p* < 0.01), and 30 (mean = 59.6%, *p* < 0.01) mM sodium lactate (*p* < 0.01) (Figure 6A). These results demonstrate a significant impact of sodium lactate supplementation on mouse embryo development.

To further examine the effects of sodium lactate on histone lactylation during mouse early embryogenesis, we first used a pan-lactylation antibody to detect lactylation levels in 2-cell embryos under 0, 5, 10, 20, and 30 mM sodium lactate. As shown in Figure 6B, nuclear fluorescence signals were observable in all groups and progressively enhanced with increasing sodium lactate concentrations, indicating elevated histone lactylation levels. The fluorescence intensities under 5–30 mM sodium lactate were extremely significantly higher than under 0 mM (*p* < 0.01). Based on the pan-lactylation antibody results, we compared the 10 mM sodium lactate group with the 0 mM control and assessed the histone lactylation changes at the 2-cell stage. In the 2-cell embryo nucleus, distinct H3K9la (1.3-fold higher than 0 mM, *p* < 0.01) and H3K14la (1.4-fold higher than 0 mM, *p* < 0.01) fluorescence was detectable in both groups, with extremely significantly stronger signals under 10 mM versus 0 mM sodium lactate (Figure 6C). Weak H3K18la and H3K56la fluorescence was observed in 2-cell embryos of both groups (Figure 6C). Additionally, clear H4K5la (1.6-fold higher than 0 mM, *p* < 0.01), H4K8la (2.2-fold higher than 0 mM, *p* < 0.01), H4K12la (1.8-fold higher than 0 mM, *p* < 0.01), and H4K16la (2.0-fold higher than 0 mM, *p* < 0.01) signals were seen in the 2-cell embryo nucleus of the 0 and 10 mM groups, with extremely significantly higher fluorescence under 10 mM compared to 0 mM sodium lactate (Figure 6D).

### 2.7. Increasing Histone Kla Levels Alters Gene Expression in 2-Cell Embryos

**Figure 7:**
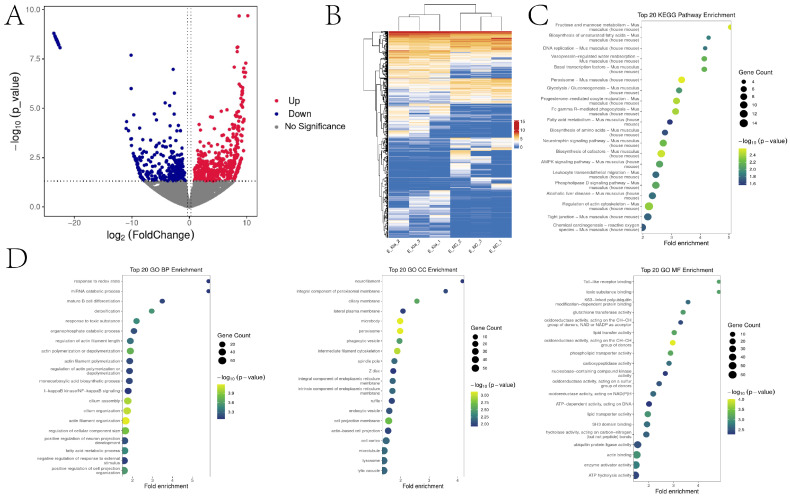
Transcriptomic changes induced by sodium lactate in mouse 2-cell embryos. (**A**) Volcano plot showing differentially expressed genes (DEGs). (**B**) Heatmap of DEG expression. (**C**,**D**) KEGG pathway and GO enrichment analyses of DEGs after 10 mM versus 0 mM sodium lactate treatment in 2-cell embryos.

To elucidate the mechanisms underlying the effects of lactylation on 2-cell embryos, we performed transcriptomic profiling of embryos treated with 10 mM sodium lactate versus CON. The results showed 494 upregulated and 394 downregulated mRNAs in the sodium lactate group compared to CON (Figure 7A,B). KEGG pathway enrichment analysis indicated that the differentially DEGs were predominantly enriched in pathways related to fructose and mannose metabolism, peroxisome, and glycolysis (Figure 7C). For biological process classification, response to redox state, organophosphate catabolic process, and actin polymerization/depolymerization were significantly enriched (Figure 7D). At the cellular component level, these proteins were highly enriched in the microbody, peroxisome, and phagocytic vesicle (Figure 7D). The DEGs were also highly enriched in molecular functions including Toll-like receptor binding, glutathione transferase activity, and lipid transporter activity (Figure 7D).

## 3. Discussion

Lactylation is a novel post-translational modification occurring intracellularly that can influence protein structure, function, and interactions [18]. Lactylated proteins play important roles in many physiological and pathological processes, including energy metabolism, cell cycle, gene expression, stress response, neurodegenerative diseases, and cancer [18]. In this study, we examined lactylation levels in different mouse tissues, and specifically investigated whether histone lactylation occurs and its dynamic changes during mouse oocyte maturation and early embryo development. First of all, our study revealed that lactylated proteins are widely present across different mouse tissues with tissue- and cell-specific distribution patterns, indicating lactylation as a common post-translational modification involved in diverse physiological processes. Then, we found that in the ovary, lactylated proteins were mainly localized in oocytes, stromal and granulosa cells, with more localization in the oocyte cytoplasm, which may be associated with oocyte growth and maturation. In uterine tissues, lactylated proteins were predominantly localized in the serosa, myometrium, and endometrium, with greater localization in the endometrium, which may relate to endometrial maintenance and blood supply during pregnancy. In testicular tissues, lactylated proteins were primarily detected in spermatogenic, spermatogonial, and testicular interstitial cells, with enriched localization in interstitial cells, which may correlate with their functions such as androgen production and regulation of spermatogenesis. During spermatogenesis, lactate is mainly produced by Sertoli cells through LDH-catalyzed glycolysis and secreted into seminiferous tubules to provide energy for spermatogenic cells [19]. The activity of LDH and lactate levels in Sertoli cells vary with the stages of the spermatogenic cycle. Decreased lactate levels in Sertoli cells can affect energy metabolism, morphology, quality, and fertilization capacity of spermatogenic cells [20]. Lactylated proteins can regulate intracellular metabolic activities and thus influence energy production and utilization.

Consistent with previous studies showing pan histone lactylation and H3K23la but not H3K18la detected from condensed chromosomes when oocytes reach MII [16], our results confirmed that histone lactylation levels were highest at the GV stage, sharply decreased at GVBD, and rebounded slightly at MII during mouse oocyte maturation, indicating dynamic changes throughout this process. This pattern is analogous to the dynamics of acetylation, methylation, phosphorylation, and other modifications that regulate DNA binding ability, chromatin remodeling, transcription factor access, and chromosome condensation during meiotic division of oocytes [4,21,22]. Moreover, our examination of specific lysine residues revealed H3K9la, H3K14la, H4K8la, and H4K12la fluorescence was strongest at GV but diminished at GVBD and MII, while H4K5la peaked at GVBD, suggesting lactylation at different sites may confer distinct functions during maturation. Analogous to our findings on lactylation dynamics, studies report changes in histone acetylation patterns like H3K18 acetylation increasing from GV to GVBD, peaking at GVBD, deacetylating at MI, and reacetylating at MII in buffalo oocytes [23]. Similarly, methylation patterns like H3K9me2 (a heterochromatin marker) tend to decrease in aged mouse GV oocytes compared to young ones [24], which could impact chromatin states and transcription. These findings highlight the intricate interplay of histone modifications like lactylation, acetylation, and methylation in orchestrating the epigenetic landscape and transcriptional programming governing oocyte meiotic maturation, with lactylation potentially complementing other modifications in this intricate regulatory system.

Lactate is an end product of glycolysis that is reused for gluconeogenesis in the liver. Recent studies have revealed a novel function of lactate in being utilized for histone lysine lactylation, which promotes the transition of macrophages from inflammatory to reparative phenotypes and activates homeostatic gene expression to maintain immune homeostasis [13,14]. The discovery of lactoyl-CoA (lactyl-CoA) in mammalian cells has indicated that lactoyl-CoA generated from glucose metabolism may constitute a potential biochemical link between lactate and histone lactylation in vivo. Our study also confirmed that total fluorescence intensities of GV and MII oocytes increased with escalating concentrations of exogenous sodium lactate, indicating sodium lactate enhances histone lactylation levels in oocytes. In GV oocytes, no significant differences in lactylation were observed at the H3K9la, H3K14la, H3K56la, and H4K5la sites between 0 and 10 mM sodium lactate, suggesting these sites are either insensitive to exogenous lactate or the effects are counterbalanced by other factors. In contrast, significant differences were detected at the H3K18la, H4K8la, H4K12la, and H4K16la sites between 0 and 10 mM sodium lactate in GV oocytes. Unlike GV oocytes, only the H3K14la and H4K12la sites showed markedly increased lactylation under 10 mM versus 0 mM sodium lactate in MII oocytes, while the other six sites remained unchanged. H3K14 and H4K12 are considered as transcription activating epigenetic markers that can increase chromatin accessibility for the binding of transcription factors and recruitment of RNA polymerases [25,26]. Therefore, we postulate sodium lactate may activate beneficial genes for oocyte maturation and development by elevating lactylation levels specifically at the H3K14la and H4K12la sites. Moreover, our study found that although sodium lactate at different concentrations exerted no significant effects on GVBD in mouse oocytes, 10 mM sodium lactate substantially improved oocyte maturation rates. This indicates that enhancing histone lactylation by sodium lactate does not markedly regulate resumption of meiosis in GV oocytes. Lactate can act as both an energy substrate and signaling molecule; hence, the effects on oocyte maturation may also involve modulation of energy metabolism and signal transduction. Our transcriptomic data suggest that altering lactylation levels affects oxidative phosphorylation in mouse oocytes, which in turn impacts reactive oxygen species levels and oocyte quality. Collectively, these results demonstrate sodium lactate may regulate histone lactylation at specific sites in oocytes, thereby influencing chromatin states, gene expression, and oocyte maturation in a site-specific manner.

In early mouse embryos, lactate is an important metabolic intermediate. Studies found that from zygotes to the 2-cell stage, mouse embryos rely heavily on glycolysis-derived lactate as the energy source, and thus have high demands for lactate. At this stage, embryos exist in a reductive metabolic state where lactate helps maintain low pH and high reduction potential, which facilitates zygotic genome activation and erasure of epigenetic modifications. After the morula stage, glucose utilization increases in embryos as they begin employing the tricarboxylic acid cycle and oxidative phosphorylation for ATP production [27]. Hence, lactate levels gradually decline while TCA cycle intermediates like α-ketoglutarate rise in embryos. Our data showed that 2-cell development rates were comparable across the five sodium lactate concentrations, indicating minimal impacts of sodium lactate at this stage. For 8-cell, morula, and blastocyst stages, development rates progressively increased from 0 to 10 mM sodium lactate and peaked at 10 mM, demonstrating appropriate sodium lactate levels can promote embryo cell division and differentiation to enhance developmental competence. This is likely attributed to elevated histone lactylation by sodium lactate, which in turn activates related signaling pathways and promotes gene expression and protein synthesis. In contrast, development rates declined from 10 to 30 mM sodium lactate, reaching the lowest level at 30 mM. This implies excessive sodium lactate inhibits embryo cell division and differentiation to impair developmental potential, either through drastic histone hyper-lactylation, which is detrimental for early embryos, or increased osmotic pressure, causing cell shrinkage.

Moreover, our research revealed that among the eight lactylation sites, H3K14la, H4K5la, and H4K12la fluorescence was detectable at all embryonic stages examined and displayed distinct changing patterns during development, suggesting differential regulatory roles of lactylation at these sites across stages. These changes may be closely related to the epigenetic reprogramming before and after zygotic genome activation, and they may participate in regulating the expression of the zygotic genome. Lactylation may alter the conformation and affinity of histone proteins, influencing the transcriptional activity of specific genes, thereby guiding the gene expression program during this critical developmental stage. Additionally, overall histone lactylation levels showed an increasing trend in 2-cell embryos, with escalating sodium lactate, especially with markedly enhanced signals at the H3K9la, H3K14la, H4K5la, H4K8la, H4K12la, and H4K16la sites. Transcriptomic data indicated that enhancing embryo lactylation altered glycolysis, which directly impacts endogenous lactate production and thus lactylation levels. As lactylation is an emerging epigenetic modification, it may play important roles in biological processes such as energy balance, oxidative stress response, and cell cycle regulation, which warrant further in-depth research. Collectively, these findings suggest sodium lactate may modulate histone lactylation at different sites to regulate the epigenetic state and expression of critical genes or transcription factors during early embryogenesis.

It is important to acknowledge some limitations of our study. First, while we observed dynamic changes in histone lactylation during oocyte maturation and embryo development, the specific mechanisms underlying the regulation of lactylation levels at different stages remain to be elucidated. Second, our study primarily focused on histone lactylation, but the potential crosstalk between lactylation and other epigenetic modifications, such as acetylation and methylation, warrants further investigation. The important message from our study is that histone lactylation plays important regulatory roles during oocyte maturation and early embryogenesis, and modulating lactylation levels can influence these processes. Our findings provide new insights into the epigenetic regulation of oocyte and embryo development, potentially leading to strategies for improving oocyte maturation rates and embryo quality. Future research should aim to elucidate the specific mechanisms by which lactylation regulates gene expression and cellular processes during oocyte maturation and embryogenesis.

## 4. Materials and Methods

### 4.1. Animals and Treatment

We obtained ICR mice (10 weeks old) for oocyte and embryo collection from SLAC Experimental Animal Co. Ltd. (Shanghai, China) and housed them in a specific pathogen-free facility. The mice were kept at a temperature of 22 ± 2 °C under a 12 h light/dark cycle and provided with unlimited access to food and water. A one-week adaptation period was allowed before commencing the experiments. All animal procedures strictly adhered to the guidelines of the Institutional Animal Care and Use Committee of Hainan University. Euthanasia of the mice was carried out after anesthesia, and various organs, including the heart, liver, spleen, lungs, kidneys, stomach, uterus, testes, muscles, ovaries, and intestine, were harvested for further analysis.

For in vivo collection of oocytes and embryos, the procedures were performed as previously described [16]. Ovaries were removed from mice, and GV oocytes were isolated by gently puncturing small antral follicles (200–300 μm) under a stereomicroscope. Embryos were obtained by flushing the fallopian tubes and uterine horns with M2 medium. Embryos at the 2-cell, 4-cell, morula, and blastocyst stages were collected at 42–44, 51–53, 84–86, and 92–94 h post-hCG injection, respectively.

To investigate the influence of sodium lactate on enhancing histone lactylation during oocyte maturation, the base medium was Opti-MEM supplemented with 4 mg of BSA. Sodium lactate solution was added with various concentrations (0 mM, 5 mM, 10 mM, 20 mM). To ensure adequate sodium lactate uptake, oocytes were initially cultured with 3-isobutyl-1-methylxanthine (IBMX, phosphodiesterase inhibitor) and sodium lactate for 24 h, after which they were transferred to the same concentration medium without IBMX for continued culture. GVBD rates were assessed at 2, 4, and 6 h after releasing inhibition, and maturation rates were determined following 14 h of culture.

To investigate the influence of sodium lactate on early embryonic development, the basal culture medium used was KSOM-AA supplemented with 4 mg of BSA and devoid of sodium lactate. Sodium lactate solutions were added in accordance with the required concentrations (0 mM, 5 mM, 10 mM, 20 mM, 30 mM). To maintain consistent osmotic pressure in the KSOM-AA culture medium with varying sodium lactate concentrations and prevent osmotic changes resulting from sodium lactate content variations, NaCl was added based on the amount of sodium lactate introduced. This ensured that the KSOM-AA culture medium with different sodium lactate concentrations possessed identical osmotic pressure.

### 4.2. Immunofluorescent Staining

The heart, liver, spleen, lungs, kidneys, stomach, uterus, testes, muscles, ovaries, and intestine were fixed and preserved in paraffin after an overnight fixation process. Tissue sections were subjected to incubation with primary antibodies (Pan la, PTM-1401, PTM Bio) at 37 °C for 2 h. Following this, the sections underwent three PBS rinses and were subsequently incubated for 1 h at room temperature with Alexa-labeled secondary antibodies (Invitrogen, Life Technologies) at a 1:500 dilution at 37 °C for 2 h. Nuclei were counterstained with DAPI (4,6-diamidino-2-phenylindole). Subsequently, the slides were visualized using a fluorescence microscope (Nikon Inc., Melville, NY, USA).

For oocytes and embryos, collected samples were fixed in 4% (*w*/*v*) paraformaldehyde (PFA) in phosphate-buffered saline (PBS) at room temperature for 20 min. Following fixation, samples underwent three washes with 1% BSA/PBS. Samples were permeabilized with 0.5% Triton X-100 and 1% BSA/PBS for 20 min, followed by three rinses with 1% BSA/PBS. The samples were blocked with 0.1% Triton X-100, 1% BSA/PBS for 30 min and incubated with primary antibodies overnight at 4 °C, including Pan la, H3K9la, H3K14la, H3K18la, H3K56la, H4K5la, H4K8la, H4K12la, H4K16la. Oocytes and embryos were incubated with secondary antibodies in the dark for 1 h and subsequently washed with 1% BSA/PBS for 5 min, repeating this process three times. Afterward, the samples were stained with DAPI for 10 min. Finally, the samples were mounted on slides, and immunofluorescence images were captured using a fluorescence microscope.

### 4.3. Western Blot

The heart, liver, spleen, lungs, kidneys, stomach, uterus, testes, muscles, ovaries, and intestine were collected and subjected to homogenization. Each well of a 12% SDS-PAGE gel received a loading of 30 micrograms of total protein for electrophoretic separation. Subsequently, the proteins were transferred onto PVDF membranes (Millipore, Bedford, MA, USA). Following the blocking of non-specific binding sites, the membranes underwent overnight incubation at 4 °C with anti-Pan la (PTM Bio, 1:1000 dilution) and anti-ACTB antibody (Proteintech Group, 1:2000 dilution). The membranes were then incubated with HRP-conjugated secondary antibodies. Finally, protein bands were visualized using Image-Pro Plus 6.0 software (Media Cybernetics, Silver Spring, MD, USA) and quantified with Quantity One software (Version 4.62, Bio-Rad Laboratories, Hercules, CA, USA).

### 4.4. RNA-Sequencing and Bioinformatic Analysis

RNA sequencing was carried out by Geekgene Co., Ltd. (Beijing, China). Total RNA was extracted to construct cDNA libraries. Following a quality assessment, the libraries were combined based on their effective concentrations and the desired data output targets. Subsequently, sequencing was performed on the Illumina NovaSeq 6000 platform. Differentially expressed genes (DEGs) were identified using the criteria of log2(fold change) > 1.2 and *p* < 0.05. To gain insights into the functional implications of these DEGs, Gene Ontology (GO) and Kyoto Encyclopedia of Genes and Genomes (KEGG) pathway analyses were conducted. The raw sequencing data were deposited in the China National Center for Bioinformation under the accession code PRJCA021062.

### 4.5. Statistical Analysis

Unless specifically indicated, all data are expressed as mean ± standard deviation (SD). Statistical analysis was performed using SPSS version 22 (IBM-SPSS Inc., Chicago, IL, USA). Variables were subjected to one-way analysis of variance (ANOVA), followed by post hoc LSD tests for group-wise comparisons. Statistical significance was considered as *p* < 0.05.

## 5. Conclusions

In this study, we have confirmed that lactylated proteins were widely detected in various mouse tissues, such as heart, liver, spleen, lung, kidney, stomach, intestine, muscle, uterus, ovary, and testis, indicating lactylation as a ubiquitous post-translational modification. During mouse oocyte maturation, histone lactylation levels significantly decreased. Supplementation of 10 mM sodium lactate in culture medium elevated both the oocyte maturation rate and histone Kla modification levels, with prominent changes at the H4K8la, H4K12la, and H4K16la sites in GV oocytes, and markedly increased Kla at the H3K14la and H4K12la sites in MII oocytes. It also affected the transcription of molecules involved in oxidative phosphorylation. Additionally, histone lactylation levels changed dynamically with embryo cell division and development during mouse early embryogenesis. Sodium lactate at 10 mM enhanced early embryo development as well as lactylation at the H3K9la, H3K14la, H4K5la, H4K8la, H4K12la, and H4K16la sites, and impacted the transcription of glycolytic molecules. This study helps reveal the roles of lactylation during oocyte maturation and embryo development, providing new insights into improving oocyte maturation rates and embryo quality.

## Data Availability

The data that support the findings of this study are available from the corresponding author upon reasonable request.
